# Occupational Injuries Among Hospital Workers: A Retrospective Study in Turkey

**DOI:** 10.3390/jcm14041050

**Published:** 2025-02-07

**Authors:** Volkan Medeni, Sultan Pınar Çetintepe, İrem Medeni, Hilal Özdemir Öztel, Fatma Bozdağ, Asiye Uğraş Dikmen

**Affiliations:** 1 Department of Occupational Medicine, Faculty of Medicine, Gazi University, Ankara 06560, Türkiye; 2 Employee Health Department, General Directorate of Public Health, Ministry of Health, Ankara 06800, Türkiye; 3 Department of Public Health, Faculty of Medicine, Gazi University, Ankara 06560, Türkiye

**Keywords:** occupational injuries, healthcare workers, working conditions, sharps injuries, workplace violence

## Abstract

**Introduction:** Occupational injuries among healthcare workers adversely affect the quality of healthcare services by undermining their physical and mental well-being. This study evaluates the frequency, characteristics, and influencing factors of occupational injuries among non-physician healthcare workers in a university hospital. **Methods:** This cross-sectional study examines occupational injuries reported between 2020 and 2023 at a university hospital in Turkey. Variables included sociodemographic characteristics, occupation, department, working hours, cause and type of injury, time of injury, affected body part, use of personal protective equipment (PPE), medical intervention, and incapacity for work. **Results:** A total of 694 occupational injuries were reported at Gazi University Hospital between 2020 and 2023, with the fewest cases occurring in 2021. Among the injured workers, 58.8% were female, 89.2% were aged between 20 and 49 years, 30.1% did not use PPE, 76.4% received medical intervention, and 11.1% experienced incapacity for work. Cleaning workers (33.6%) and nurses (32.1%) accounted for the highest proportion of injuries. Sharps injuries were the most common type (48.8%), while injuries to the hands, fingers, and wrists comprised 53.3% of cases. The highest frequency of injuries occurred between 11:00 and 11:59 am. Sharps injuries were significantly associated with gender, age, educational background, occupation, working hours, and injury time. In contrast, cases of workplace violence were significantly associated with gender, occupation, and working hours. **Conclusions:** Sharps injuries and violence represent critical occupational hazards. Preventive strategies should be tailored to healthcare workers’ occupational roles and educational levels. Effective surveillance systems and reporting mechanisms should be implemented to promote workplace safety and reduce the risk of injuries.

## 1. Introduction

An occupational injury is an unplanned and unexpected event occurring due to a person’s work that may result in injury, illness, or death [1]. According to the “Law on Occupational Health and Safety” numbered 6331, which came into force in 2012 and was updated in 2015, occupational injuries are events that occur in the workplace or during the execution of work, leading to death or compromising physical or mental integrity. Such injuries must be reported to the Social Security Institution within three working days by workplaces and within ten working days by healthcare service providers [2]. Employees in healthcare institutions who are required to report these incidents constitute a high-risk group for occupational injuries. Healthcare workers are exposed to numerous risk factors in their work environment, many of which can cause injuries. Non-physician health workers, who are nurses, health technicians, laboratory technicians, medical and non-medical technicians etc., play a vital role in supporting the sustainability of health services with unique job descriptions and working conditions. Their physical engagement and diverse work environments may increase their risk of occupational injuries. Examining this group is crucial for accurate risk assessments. Common occupational injuries among healthcare workers include sharps injuries, slips, trips, falls, collisions, workplace violence, harassment, traffic accidents, electric shocks, chemical exposure, and fires [3,4]. “Sharps” include needles and items such as scalpels, lancets, razor blades, scissors, wires, retractors, clamps, pins, staples, cutters, and glass items. Any object that can cut the skin can be considered “sharp” [5].

Hospitals and other healthcare organizations are the second most hazardous workplaces for non-fatal injuries, following industrial areas [6]. Such injuries can adversely affect healthcare workers’ physical and mental health, increasing the likelihood of errors and subsequent accidents. A study in 2022 showed that the presence of physical violence, verbal harassment, poorer perceived workplace culture, and poorer perceived leadership support among healthcare workers led to more suicidal ideation [7]. These factors compromise patient safety and diminish the quality of healthcare services. Additionally, occupational injuries result in direct and indirect economic losses due to treatment costs and workforce absenteeism [8]. Sharps injuries, in particular, pose significant risks for infectious diseases such as Hepatitis B, Hepatitis C, and HIV [9,10].

A systematic review of occupational injuries among healthcare workers in 2018 revealed that three-fifths of workers had experienced an injury during their careers, while two-fifths reported an injury within the past year [11]. A 2024 study in Makkah, Saudi Arabia, determined that nurses (64%) are the most common group among healthcare workers who experience workplace injuries [12]. In a study examining compensation claims for occupational injuries, healthcare workers reported increased incidents during the COVID-19 pandemic despite decreases in other professions. During this period, overexertion was the most frequently reported non-COVID-19 occupational injury, followed by sprains, tears, and strains. Between 2019 and 2021, there was a decline in non-COVID-19-related injuries but an increase in stress-related mental health disorders and injuries due to physical impact [13]. According to occupational injury statistics in Turkey, the incidence of such injuries between 2019 and 2023 ranged from 2.32 to 2.96 per 1000 workers, with an upward trend starting in 2020 [14]. These statistics primarily reflect private and self-employed workers and do not account for the public sector, where most healthcare workers are employed. Consequently, the available data likely needs to be more representative of the true prevalence of occupational injuries in the healthcare sector.

In Turkey, no official data comprehensively evaluates occupational injuries among hospital staff by occupational groups and working environments. Moreover, existing data must address the factors influencing the causes and consequences of these injuries. Studies examining all types of occupational injuries involving hospital workers in Turkey during and after the pandemic are notably limited. Most research in Turkey tends to focus on specific occupational groups or particular types of occupational injuries. Therefore, local-scale studies are invaluable for mitigating the harmful effects of occupational injuries on healthcare workers, institutions, and society. In this context, our study aimed to examine the frequency of occupational injuries among non-physician healthcare workers in a university hospital, along with their associated characteristics and influencing factors.

## 2. Methods

As of the end of 2023, 3436 non-physician healthcare professionals were employed at Gazi University Faculty of Medicine Hospital, which is where this study was conducted. Data for this cross-sectional study were obtained from the notification forms of all occupational injuries reported to the hospital’s Occupational Health and Safety Unit between 1 January 2020, and 31 December 2023. Staffed by two occupational safety specialists, this unit is responsible for recording, registering, and managing statistical activities related to occupational injuries. The forms applied during the study process were standardized and included all hospital-related events reported by non-physician healthcare workers. The form was prepared based on the “SGK-032-Occupational Accident and Occupational Disease Notification Form” prepared by the SSI institution. The data in the study is based on a self-reported format, therefore some questionnaires have some missing values. The forms kept manually were transferred to a computer environment, and a database was prepared as an Excel file.

When an occupational injury occurs in the hospital, the affected employee is referred to the Occupational Health and Safety Unit after receiving medical interventions. While the unit was established in 2014, it has operated comprehensively since 2020. Physicians, being academic staff, follow a different process for reporting occupational injuries; their notifications are handled by an independent unit within the deanery and are not included in the scope of this study. Physicians typically perform specialized tasks such as diagnosing, planning treatments, and managing patient care in controlled environments following specific protocols. Their work settings involve fewer physical risks, and professional development programs often help prevent occupational injuries. However, studies on occupational injuries have predominantly focused on physicians, highlighting the need for research on non-physician healthcare workers [15,16].

The variables analyzed in this study included age, gender, height, body weight, marital status, educational background, occupation, department, working hours, cause and type of injury, affected body part, time and day of injury, starting time on the day of injury, use of personal protective equipment (PPE) at the time of injury, medical intervention following injury, and the presence of incapacity for work.

The body mass indexes (BMIs) of the injured individuals were categorized as follows: underweight (<18.50 kg/m^2^), healthy weight (18.50–24.99 kg/m^2^), overweight (25.00–29.99 kg/m^2^), and obese (≥30.00 kg/m^2^). Injured body parts were grouped into the following categories: hand, wrist, and finger; foot, ankle, and toe; leg, knee, and hip; head, neck, and face; eye; arm, elbow, and shoulder; trunk and waist; multiple body parts; and internal organs.

The distribution of occupational injuries was analyzed by year (2020–2023) and month (January–December). The times of occupational injuries were analyzed over a 24-h period, while working hours were analyzed over a 16-h period.

The causes of injuries were categorized into unsafe behaviors, unsafe environments, and other causes. Unsafe behavior occurs when individuals intentionally deviate from their organization’s safety rules, procedures, and standards [17]. The work environment, including physical spaces, equipment, and materials used during duties, plays a key role in workplace safety outcomes. A safe working environment depends on and encourages safe employee behaviors [18,19]. Unsafe behaviors included recapping needles, failing to use personal protective equipment, lifting excessive loads, improper waste segregation, sudden employee movements, and performing tasks outside assigned responsibilities. Unsafe environments included insufficient physical space, using defective tools or personal protective equipment, slippery floors, and inadequate warning signs. Other causes included exposure to physical or verbal violence, sudden movements by patients or their relatives, and patient unconsciousness.

For group comparisons, physical and verbal violence were combined and analyzed under the category of violence. Educational levels were grouped as “high school and below”, including those who did not complete school and primary, secondary, and high school graduates, and “associate degree and above”, including associate’s, bachelor’s, and master’s degree graduates. Total working time in the hospital was categorized into “less than five years” and “five years or more”, while total working time within the department was divided into “less than one year” and “one year or more”. The time intervals of the shifts were based on the hospital working pattern, and the injury times were analyzed as 08:00–16:59, representing the day shift, and 17:00–07:59, representing the night shift. For comparisons between occupations, employees other than nurses and cleaners—the largest occupational groups—were grouped under “other”. Certain categories were combined under broader headings to ensure an adequate sample size for statistical analysis, following the categorization approaches used in similar studies. More detailed classifications of the variables would have resulted in insufficient group sizes, limiting the reliability of the analyses. This grouping also aligns with the analysis’s assumptions and enhances the results’ validity.

Ethical approval for the study was obtained from the Gazi University Ethics Commission (2023-1477). Statistical analyses were conducted using the Statistical Package for Social Sciences. In descriptive findings, categorical variables were presented as numbers and percentages. Comparisons between groups were made using Pearson’s chi-square test, Yates’ continuity correction, and Fisher’s exact probability test. Statistical significance was set at *p* < 0.05.

## 3. Results

Between 2020 and 2023, 694 occupational injuries occurred at Gazi University Medical Faculty Hospital. Among the injured employees, 58.8% were women, 52.2% were married, and 89.2% were aged between 20 and 49. Regarding body mass index, 37.8% of the injured employees were overweight, while 17.4% were obese. Regarding educational background, bachelor’s degree holders represented the largest group, comprising 36.2% of the injured. Cleaning workers (33.6%) and nurses (32.1%) were the two occupational groups with the highest number of injuries. Occupational injuries were most frequently reported in wards (26.9%), intensive care units (14.8%), and emergency services (11.5%). Employees with a total working period of less than two years in the hospital accounted for 34.4% of the injuries, and those with less than two years of work experience in their department represented 60.1% of cases (Table 1).

Unsafe behavior was responsible for 57.3% of occupational injuries, while 30.1% were attributed to unsafe environments. Injuries caused by cutting and piercing tools constituted 48.8% of all cases, and injuries to the hand, fingers, and wrist accounted for 53.3%. Among those injured, 69.9% were using personal protective equipment, 76.4% received medical intervention, and 88.9% did not experience incapacity for work following the incident (Table 2).

Sharps injuries were primarily caused by needle sticks, accounting for 59.6% of cases, often occurring during blood collection, injections, or treatment procedures. Another 20.9% resulted from waste collection and transportation, including medical and sharps waste handling. The remaining 19.5% were attributed to instrument sterilization, laboratory work, and repair procedures.

**Chart 1:**
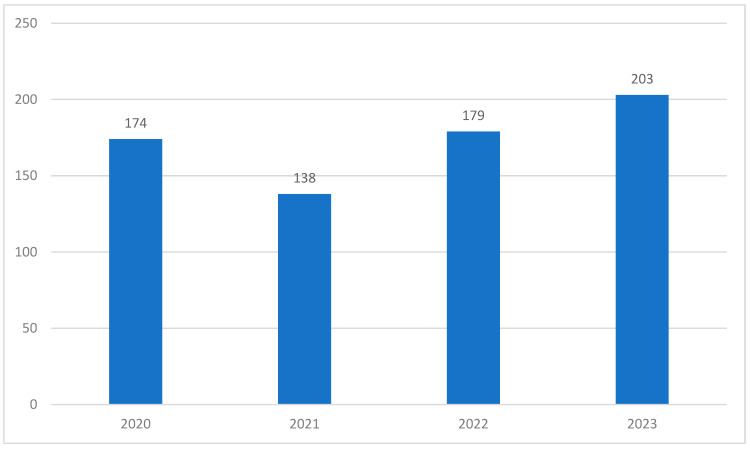
Distribution of the occupational injuries by years, Turkey, 2020–2023. (*n* = 694).

**Chart 2:**
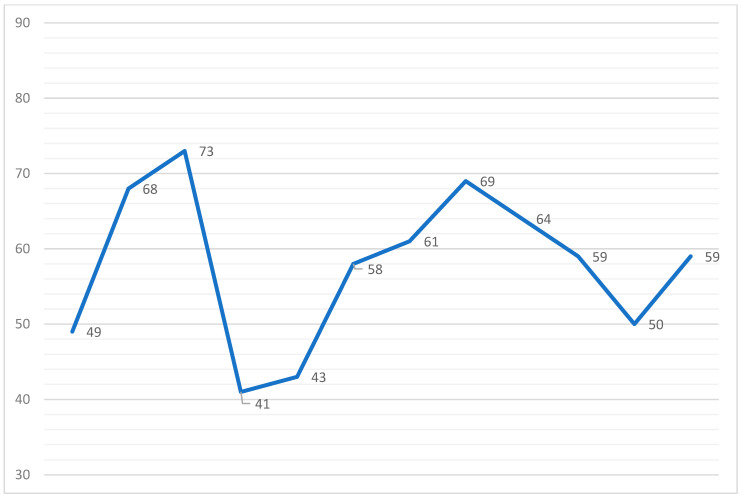
Distribution of the occupational injuries by months, Turkey, 2020–2023. (*n* = 694).

The number of occupational injuries varied over the years, starting at 174 in 2020, decreasing to 138 in 2021, and then rising to 179 in 2022 and 203 in 2023 (Chart 1). Monthly trends revealed that the highest number of injuries occurred in March (73 cases), followed by a sharp decrease in April (41 cases) and May (43 cases). A secondary peak was observed in August (69 cases), with a continued rise during the summer months (Chart 2).

**Chart 3:**
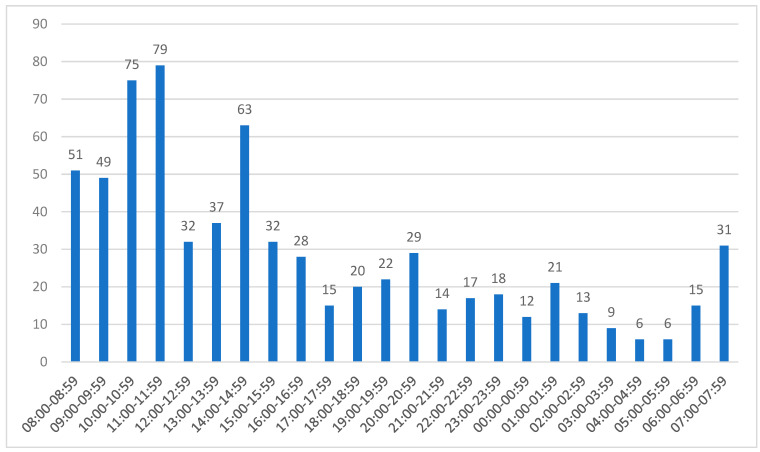
Distribution of the occupational injuries by hours, 2020–2023. (*n* = 694).

**Chart 4:**
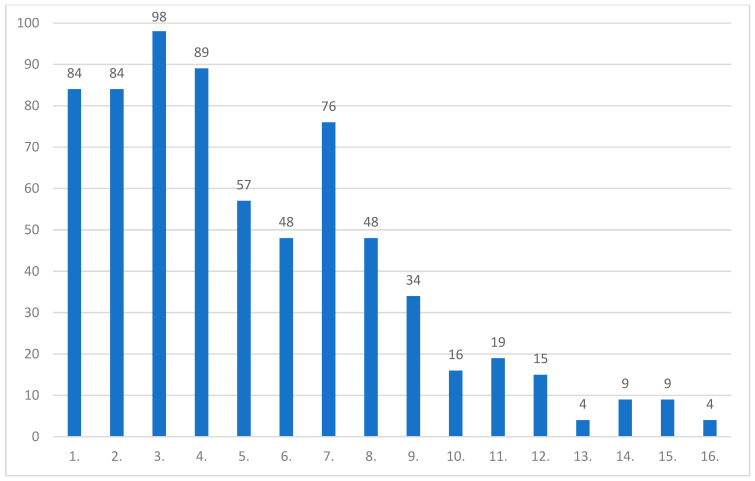
Distribution of the occupational injuries by working hours, 2020–2023. (*n* = 694).

The hourly distribution of injuries showed a concentration during specific times of the day, with 79 injuries occurring between 11:00–11:59, 75 injuries between 10:00–10:59, and 63 injuries between 14:00–14:59 (Chart 3). Analysis by working hours revealed that the first four hours of work were particularly hazardous, with 84, 84, 98, and 89 injuries recorded, respectively (Chart 4).

Gender-based analysis showed that sharps injuries were more common among women (54.7%), while men experienced higher rates of violence (13.6%). Sharps injuries decreased with age, from 57.1% in employees under 30 to 30.8% in those aged 50 and older, and were most frequent among nurses (70.9%) and workers with higher education (59.5%). Violence exposure was higher among those with five or more years of hospital experience (14.2%) and varied significantly by gender, occupation, and work experience. Night shift workers reported more sharps injuries and violence than day shift workers. Both sharps injuries and violence showed statistically significant differences based on factors such as age, gender, education, occupation, and work duration (Table 3).

Statistically significant relationships were found between the sharps injury type and factors such as gender, age, educational status, occupation, and injury time. Among female employees, 64.6% of injuries were caused by needles, 24.7% by waste collection, and 10.8% by other causes, whereas for male employees, 49.1% of injuries were due to waste collection and 25.9% to needles. Needlestick injuries were reported by 57.3% of employees under 35 and 42.1% of those aged 35 and older. Waste collection injuries were more frequent among employees with a high school education or lower (64.7%), while 89.0% of injuries among those with an associate degree or higher were needle-related. Nearly all injuries among nurses (97.5%) were caused by needles. Additionally, 45.8% of sharps injuries occurred during 08:00–16:59 and 58.5% of the injuries that occurred during 17:00–07:59 were needle-related (Table 4).

## 4. Discussion

Our cross-sectional study examined occupational injuries experienced by healthcare workers in a university hospital in Turkey during and after the pandemic.

In our analysis of the educational levels of employees who experienced occupational injuries, we found that the highest frequency was among bachelor’s degree graduates, followed by high school graduates. Another study conducted in Turkey found that those with a master’s degree or above experienced more occupational injuries. [20]. Similarly, a study conducted in another university hospital in Turkey found that bachelor’s degree graduates experienced the highest frequency of occupational injuries in the preceding year [21]. In Brazil, more than half of the healthcare workers who experienced occupational injuries were high school graduates or higher [22]. In a study conducted in Ethiopia, four out of every five healthcare workers who experienced occupational injuries were university graduates or higher [23]. These findings highlight the influence of national workforce compositions on injury statistics. In Turkey, a substantial proportion of hospital workers have a bachelor’s degree or higher, making this group the majority at risk. Conversely, in countries like Brazil, healthcare activities are predominantly carried out by professionals with high school-level education, resulting in this group being the most affected by occupational injuries. The relatively high frequency of injuries among employees with advanced education levels in Turkey suggests that these groups may face greater exposure to specific occupational risks. The hospital where our research was conducted is a university hospital. Working conditions in university hospitals might require more personnel with undergraduate or higher education levels. Highly educated staff may often focus more on the technical aspects of their work, which might lead to overlooking safety procedures. Additionally, individuals with higher education levels might pay less attention to routine safety training and procedures as they become more integrated into their roles. Balancing numerous responsibilities is also likely to cause challenges in time management, leading to distraction and an increased risk of injuries. In addition, we consider that the emphasis on teaching and research activities in university hospitals may potentially increase the likelihood of injury by encouraging innovative or trial-and-error approaches. Understanding the educational background of staff, identifying those who still need to complete training, and knowing the frequency of injuries among all healthcare staff is essential for planning future interventions.

In our study, cleaning personnel and nurses emerged as the most frequently injured occupational groups. This finding is consistent with other studies, including one conducted in a Turkish hospital that excluded cleaning staff, where nurses were identified as the most common victims of occupational injuries [24]. Internationally, similar findings have been observed. Studies in Ghana and Sweden also identified nurses as the occupational group most affected by injuries [25,26]. In Jordan, a six-year analysis of needle stick and sharps injuries revealed that nurses and cleaning personnel were the most frequently injured healthcare workers [27]. Likewise, a study conducted in Brazil found that nurses accounted for the most occupational injuries, with cleaning personnel ranking second [28]. Several factors likely contribute to this trend. Healthcare workers, mainly cleaning staff and nurses, face significant risk factors in their daily routines. Nurses may frequently handle sharp instruments during invasive procedures such as injections, blood draws, and IV insertions, often in high-stress, fast-paced environments. Long working hours, heavy patient loads and emotionally demanding situations can distract them while working; it is possible that this increases the likelihood of injury. Similarly, cleaning personnel face workplace exposure to chemical cleaning agents that could cause skin irritation, poisoning or burns, and serious respiratory problems if inhaled. We believe that physically demanding tasks such as lifting heavy equipment or carrying loads may contribute to back pain and musculoskeletal injuries, while wet floors increase the risk of slips, falls and collisions. Moreover, the improper use of personal protective equipment when handling infectious materials, such as blood or bodily fluids, may further elevate the risk of infections. In our hospital, both nurses and cleaning personnel are heavily involved in patient care activities, frequently handle sharp and piercing instruments, and are exposed to significant workloads. Additionally, the underreporting of occupational injuries among nurses working in our hospital may be a factor in our findings. It could be due to heavy workloads, lack of time, or the perception that specific incidents are not severe enough to warrant reporting. It is important to implement actions such as enhancing safety measures for these groups, promoting the use of personal protective equipment, and ensuring the sustainability of inspections aimed at minimizing environmental risk factors.

This study found that the most common occupational injuries occurred in the inpatient ward, intensive care unit, and emergency department. Similarly, a study conducted in Turkey identified the inpatient ward, intensive care unit, and operating room as the top three departments where healthcare personnel with occupational injuries worked [29]. A study conducted in China found that general wards, operating rooms, intensive care units, disinfection supply centers, and outpatient clinics were the top five locations where healthcare workers were exposed to sharp injuries [30]. A study analyzing occupational injuries among cleaning personnel in a university teaching hospital in Ethiopia reported that the intensive care unit, inpatient ward, and emergency department were the most common sites of such incidents [31]. A study conducted in Qatar identified operating rooms, inpatient wards, and emergency departments as the most common locations for injuries [32]. Factors such as high patient volume, frequent emergency interventions, and the widespread use of sharps in these departments may explain these findings. Furthermore, with the intense and stressful working environments in these settings, factors such as increased unsafe behavior and a weak safety culture may heighten risk factors for occupational injuries, such as carelessness and fatigue.

In our study, more than half of the reported injuries were caused by unsafe behavior. Other studies conducted in Turkey (2016) and Portugal (2012) also identified unsafe behavior as the leading cause of occupational injuries, with the majority of incidents attributed to this factor [23,24,25,26,27,28,29,30,31,32,33,34,35]. In his analysis of approximately 75,000 injuries, Heinrich (1979) found that 88% of industrial injuries were primarily caused by unsafe behavior [36]. Similarly, Fleming and Lardner (2002) reported that unsafe behaviors by employees accounted for nine out of ten occupational injuries and near-misses [37]. Healthcare workers are particularly vulnerable to exhibiting unsafe behavior due to factors such as long working hours, heavy workloads, and workplace stress, including mobbing. However, the focus on controlling hazards should not solely target employee behavior. The underlying causes of unsafe behaviors are often systemic issues, such as inadequate occupational health and safety training, insufficient workplace inspections, low motivation and concentration, miscommunication, inexperience, or weaknesses in safety culture. Addressing these root causes is essential to creating safer working environments. Incorporating measures such as increasing and regularizing staff training programs aimed at reducing unsafe behaviors, improving safety culture, and enhancing hospital inspections will contribute to reducing occupational injuries.

When examining the types of injuries in this study, cutting and piercing tool injuries accounted for approximately half of all occupational injuries. Additionally, one in ten workers reported experiencing violence as a type of injury. Another study conducted in Turkey found that more than half (59.4%) of the injuries were caused by sharp and penetrating instruments [38]. Similarly, an eight-year study conducted in Brazil (2019) found that sharps were the most common cause of hospital injuries [39]. In South Korea, research revealed that 7.7% of occupational injuries among medical service providers were caused by violence [40]. Cultural, social, and workplace factors likely influence the variation in findings across different studies. Differences in healthcare systems, working conditions, training programs for healthcare workers, perceptions of violence, and reporting methods may all contribute to these disparities. Despite these variations, it is evident that sharps injuries are a critical issue in occupational safety, and violence continues to pose a significant threat to the well-being of healthcare workers. Our study also found that over half of the participants experienced hand, wrist, or finger injuries. Similarly, another study in Turkey (2016) identified the hand and arm region as the most commonly injured body parts [41]. A study conducted in India during the COVID-19 pandemic found that the most common occupational injuries occurred in the fingers [42]. Hands are particularly susceptible to injuries due to their extensive use in work processes, which may explain why sharps injuries are the most common type of occupational injury. Additionally, factors such as the lack of appropriate PPE and insufficient safety measures likely contribute to the high frequency of hand injuries. This may suggest that healthcare workers should receive regular training and periodic supervision on the correct and safe use, maintenance, and cleaning of instruments. During the pandemic, increased stress among healthcare workers affected occupational injuries. Programs aimed at enhancing stress management and awareness of safety should be considered. In addition to promoting personal protective equipment, we consider that innovative prevention strategies such as needle-free systems, cut-resistant sensor gloves, and advanced disposal methods should be implemented to minimize risks. AI systems that analyze potential risks, warn workers in hazardous situations, and create risk maps could be expanded in healthcare settings. Ergonomic workspaces, clear and safe protocols for handling sharp instruments, and technological, organizational, and strategic solutions are essential actions to prevent occupational injuries.

In this study, one out of every three employees who experienced occupational injuries reported not using PPE. Similarly, a study conducted in Turkey found that more than one-fourth of hospital workers who sustained occupational injuries admitted to not using PPE at the time of the incident [43]. A study from Brazil (2019) reported that nearly one-fourth of healthcare workers who suffered injuries did not use PPE [44]. In Saudi Arabia, research revealed that over one-third of participants acknowledged inconsistent use of PPE, with this behavior being linked to a higher risk of occupational injuries [45]. These findings suggest that a lack of a fully established occupational health and safety culture in these countries, combined with insufficient emphasis on the importance of PPE, may account for the observed similarities.

In our study, approximately one in ten participants reported experiencing incapacity for work following an occupational injury. A five-year retrospective study conducted in Turkey similarly found that 10% of healthcare workers became incapacitated for at least one day due to occupational injuries [46]. In Brazil, three-tenths of hospital workers who sustained injuries required leave following the incident [47]. Given that most hospital-related injuries involve sharps, it is unsurprising that the frequency of injuries resulting in incapacity is relatively low. This observation aligns with the accident pyramid theory developed in the early 2000s. According to this theory (2018), for every workplace death, there are approximately 30 injuries leading to lost workdays, 300 injuries that do not result in lost workdays, 3000 near misses, and 300,000 risky behaviors [48]. In our study, the 9-to-1 ratio of injuries not resulting in lost workdays to those that did support this theory significantly.

When evaluating the number of occupational injuries over the years in our study, we observed a noticeable decrease in 2021. This decline can likely be attributed to the COVID-19 pandemic. Turkey detected its first case of COVID-19 on 11 March 2020. Authorities imposed a curfew on April 1, 2020, and gradually began normalizing the situation in the following months. The year 2021 saw the most severe effects of the pandemic in Turkey, including a total lockdown in April and May. During this period, hospital admissions decreased, except for emergencies. Our study found that the months with the fewest occupational injuries over the four-year period were April and May. Although the pandemic increased stress among healthcare workers due to the outbreak, our study found that the lockdown during these months likely led to fewer hospital admissions, resulting in a decrease in the number of occupational injuries. Additionally, visitor access to hospitals was restricted during the pandemic, reducing the contact between hospital staff, patients, and their relatives. This reduction in interpersonal interactions may have decreased the risk of occupational injuries by lowering the overall workload in hospitals. The Ramadan period also coincided with April and May during the study. During Ramadan, changes in working hours, lunch breaks, and rest periods were implemented across departments, contributing to the decrease in occupational injuries. Due to the pandemic, non-urgent surgeries and routine health services were postponed, and individuals avoided going out to reduce the risk of contracting COVID-19. The curfews further reduced hospital admissions. Additionally, some hospital staff, particularly administrative and support workers, transitioned to remote work, which naturally reduced the risk of injuries in the physical environment. Furthermore, health and safety protocols were strengthened in hospitals to prevent the spread of COVID-19, and regular use of personal protective equipment likely also contributed to the reduction in occupational injuries.

When analyzing the distribution of occupational injuries by time, our study found that injuries occurred most frequently between 10:00 and 11:59 and during the first hours of work. A similar study in Turkey also found that occupational injuries occurred most frequently between 08:01 and 12:00 [24]. A Uganda study found that healthcare workers’ occupational injuries occurred most frequently in the afternoon (2 p.m.–7 p.m.) [49]. Similarly, a study in Colombia found that occupational injuries in a university hospital were most common between 10:00 and 12:00 [50]. Morning hours are typically the busiest time in hospitals, and this increased workload may lead to reduced attention and energy levels, making employees more prone to injuries. The stress associated with this heightened activity can increase the likelihood of mistakes. In addition, employees may rush to complete tasks in order to take their lunch breaks, leading to distraction and neglect of safety precautions. Fatigue and hunger during this time can also heighten the chances of errors. To prevent such situations, shift times or hours could be adjusted based on the known busy periods of the hospital, or rotating shifts could be developed. Additionally, by increasing or decreasing the number of staff according to hospital demand, a more equitable workload distribution could be organized, potentially reducing the frequency of injuries. In this study, sharps injuries were more common among female nurses with a bachelor’s degree or higher. A study conducted in Iran found that a bachelor’s degree increased the risk of needlestick injury by 1.41 times, and a master’s degree increased it by 2.25 times compared to those without academic education [51]. Studies in Saudi Arabia also found that female nurses experienced 2–3 times more sharps injuries than male nurses [52,53]. Possible reasons for the higher incidence of sharps injuries among nurses with higher education include their involvement in complex and risky procedures, intense work tempos, and greater responsibilities in clinical practice. The higher risk of injury in women compared to men may be due to female nurses primarily performing tasks related to blood collection and patient care, while male nurses tend to work in administrative or technical roles with less direct patient contact. In the study, the gender percentages were examined by profession, and the gender concentration in each group was identified. Based on this, targeted training or organizational interventions can be implemented for those groups.

Our study also found that the frequency of sharps injuries decreased with age, years of work in the hospital, and years of work in the department. Another study conducted in Turkey found that occupational injuries decreased with age [29]. Similarly, a study conducted in Canada identified a decrease in occupational injuries with age [54]. Similarly, a study in Saudi Arabia found that the prevalence of needlestick injuries was 5.5% among participants over 50 years old, compared to 13.1% in those aged 20–29 [55]. A study conducted in Iraq showed that injuries increased with age and years of work experience [56]. These discrepancies may be due to various confounding factors. More experienced healthcare workers may have developed better professional skills and techniques, making them more cautious when handling sharps. As age and experience increase, workers may become more aware of occupational safety and apply necessary precautions more carefully. On the other hand, younger workers may benefit from modern safety protocols and training. While younger healthcare workers often have better physical skills and reflexes, allowing them to respond more quickly to sudden hazards, this advantage can sometimes be offset by a lack of experience. Further research in different hospitals is needed to better understand the relationship between age, experience, and sharps injuries.

According to our findings, male hospital employees were more likely to experience violence than female employees. In hospitals, male workers may occupy positions that require physical strength and carry higher risks, such as those in departments where violent incidents are more common. As a result, they may encounter aggressive or violent patients and visitors more frequently. Additionally, we hypothesize that some cultural and social norms may create the perception that men are better equipped to cope with violence or that they are expected to face violent situations more often. This perception could make male workers more likely to be targeted in violent incidents. Some patients or their relatives may perceive male healthcare workers as more substantial and more resilient, which may lead violence being directed more towards male employees. Professions like security and cleaning tend to have more male workers, who may face more physically demanding situations. Additionally, female healthcare workers may sometimes receive more empathy due to societal gender roles. The belief that men are more resilient to or capable of handling violence could contribute to this behavior being directed toward male workers. To prevent such situations, healthcare workers should receive training on coping strategies for both physical and emotional/psychological violence. The role of gender in the workplace should also be addressed, and awareness should be raised about the relationship between gender and violent or aggressive behaviors. Hospitals should implement panic buttons, security cameras, and safe spaces and strengthen security measures. Healthcare workers should regularly receive training on effective communication with patients and relatives. Hospitals should establish anti-violence policies and systematically report and follow up on incidents of violence.

In this study, night shift workers experienced more sharps injuries than day shift workers. Similar studies conducted in Turkey (2016) and Ethiopia (2016) also found that night shift workers had more sharps injuries [57,58]. A study conducted in Iran (2006) identified working the night shift as the most significant risk factor for needlestick injuries [59]. Disruption of the natural biological rhythm during night hours reduces concentration and prolongs reaction times. Additionally, since fewer personnel are typically available during night shifts, the workload may increase, and the combination of increased workload, fatigue, and distraction may elevate the risk of injury. To address this situation measures such as adjusting shifts to allow for rotating schedules, organizing shift hours with shorter intervals considering fatigue, and increasing the number of staff during night shifts should be implemented.

This study has several limitations. First, the data were collected only from non-physician healthcare workers at a university hospital, so the findings may not be applicable to other healthcare settings. Collecting data only from non-physician staff prevents a comprehensive analysis of all healthcare workers. If injuries experienced by doctors and other professionals are overlooked, a complete picture of the risks faced by all workers in the healthcare sector will not be presented. However, analyzing occupational injuries between 2020 and 2023 in our study allows for evaluating trends over time, providing more robust results compared to data from a single period. Another limitation is that our study only considered reported cases of occupational accidents, which may have led to incomplete data as some injuries may not have been reported (e.g., verbal violence, minor injuries). Healthcare workers may believe reporting every occupational injury could lead to negative consequences, which may cause hesitation in reporting occupational injuries. The perception of employees who report injuries as weak is another factor driven by workplace culture and social norms. These emotional and psychological barriers can influence healthcare workers’ decisions to report occupational injuries, thus creating a significant obstacle to accurately recording injuries and implementing proper safety measures. Underreporting of injuries results in an inaccurate representation of actual accidents, leading to misleading conclusions about the proper frequency and variety of incidents. It can lead to incomplete and misleading decisions when determining preventive measures and establishing occupational safety policies. Additionally, some information may have been based on subjective statements during data collection. Nonetheless, since the study data were gathered from the occupational injury notification forms regularly maintained by the occupational health and safety unit, the data were collected systematically and consistently.

It is challenging to isolate the impact of data deficiencies on the results quantitatively and to assess the frequency of unreported injuries objectively and whether these omissions are systematic. Therefore, addressing the impact of these limitations on the overall validity and reliability of the study requires a more comprehensive data collection approach and a larger sample size. To encourage accurate reporting, strategies such as providing a safe reporting environment for employees, ensuring the confidentiality of the reporting process, and informing staff that reporting will not lead to negative consequences can be implemented. Additionally, to enhance the reliability of self-reported data, cross-checking this information with objective and independent sources, such as hospital records or insurance data related to occupational injuries, would improve the accuracy and validity of the findings.

## 5. Conclusions

This cross-sectional study examined occupational injuries among healthcare workers during and after the pandemic. Our findings indicate that occupational injuries vary based on gender, age, education level, occupation, work duration, and shift. Sharps injuries and violence are particularly concerning, posing significant risks to healthcare worker safety. Measures should be tailored to shift schedules, job roles, and education levels to mitigate these injuries. Further research is needed to enhance the occupational health and safety culture, ensure regular use of personal protective equipment, and identify risks.

This study fills an important gap by focusing on a group of healthcare workers that is generally less emphasized: non-physician healthcare workers. Non-physician healthcare workers play a critical role in healthcare services. However, research on occupational injuries among them could be more extensive. Our study contributes significantly to the literature in this field by examining the risks these workers face and the occupational injuries they experience.

Additionally, examining the trends in occupational injuries among healthcare workers during and after the pandemic is another unique contribution of our study. The pandemic caused a significant increase in workload and psychological pressures in the healthcare sector, which may have increased injury rates. Our study provides valuable insights into how the changing working conditions during and after the pandemic have affected the types and characteristics of occupational injuries experienced by healthcare workers. The findings, particularly regarding how factors such as heavy workload, personal protective equipment use, and increased stress have shaped injury risks, offer valuable information for future preventive measures and health policies.

An effective surveillance system for tracking and analyzing occupational injuries is essential for identifying risks and implementing preventive measures. Employees should be encouraged to report injuries while ensuring their confidentiality. Hospitals should organize awareness programs and provide regular occupational health and safety training. Health institution managers should implement policies that create safe working environments and regularly assess and update these policies to improve safety. This approach will help protect healthcare workers and ensure the delivery of quality healthcare services.

## Figures and Tables

**Table 1:** Characteristics of healthcare workers with occupational injuries, 2020–2023.

	Number (*n*)	Percentage (*%*)
**Gender**		
Female	408	58.8
Male	286	41.2
**Age groups**		
Under 20 years	10	1.4
20–29 years	265	38.2
30–39 years	191	27.5
40–49 years	163	23.5
50 years and above	65	9.4
**Body-mass index**		
Underweight	20	2.9
Healthy weight	291	41.9
Overweight	263	37.8
Obese	121	17.4
**Marital status**		
Married	362	52.2
Single	332	47.8
**Educational status**		
No school	3	0.4
Primary school	115	16.6
Middle school	58	8.4
High school	187	26.9
Associate degree	59	8.5
Bachelor’s degree	251	36.2
Master’s degree	21	3.0
**Occupation**		
Cleaning staff	233	33.6
Nurse	223	32.1
Caregiver	99	14.3
Health technician	42	6.1
Medical Secretary	30	4.3
Technical staff	21	3.0
Security guard	20	2.9
Paramedic	11	1.6
Laborant	11	1.6
Other staff	4	0.6
**Total working time in hopital**		
Less than 1 year	102	14.7
1–2 years	137	19.7
3–5 years	116	16.7
6–9 years	104	15.0
10–19 years	152	21.9
20 years and more	83	12.0
**Department**		
Wards	187	26.9
Intensive care units	103	14.8
Emergency services	80	11.5
Hallway	74	10.7
Outpatient clinics	44	6.3
Ambulatory treatment units	42	6.1
Outside the hospital	34	4.9
Operating rooms	33	4.8
Imaging units	26	3.7
Blood collection units	26	3.7
Laboratories	25	3.6
Administrative units	20	2.9
**Total working time in department**		
Less than 1 year	204	29.4
1–2 years	213	30.7
3–5 years	121	17.4
More than 5 years	156	22.5

**Table 2:** Characteristics of occupational injuries, 2020–2023.

	Number (*n*)	Percentage (*%*)
**Occupational injury cause**		
Insecure behavior	398	57.3
Insecure environment	209	30.1
Other *	87	12.5
**Occupational injury type**		
Sharps injury **	339	48.8
Slips, trips, and falls	88	12.7
Collision	62	8.9
Compression, crush	40	5.8
Verbal violence	36	5.2
Physical violence	32	4.6
Chemical causes	26	3.7
Sprain, strain	22	3.2
Blood and body fluids exposure	21	3.1
Animal attack	11	1.6
Burn	10	1.4
Other ***	7	1.0
**Personal protective equipment use**		
No	209	30.1
Yes	415	69.9
**Medical intervention**		
No	164	23.6
Yes	530	76.4
**Incapacity for work**		
No	617	88.9
Yes	77	11.1
**Injured body region**		
Hand, wrist, finger	370	53.3
Foot, ankle, toe	56	8.1
Leg, knee, hip	48	6.9
Head, neck, face	46	6.6
Eye	42	6.1
Arm, elbow, shoulder	35	5.0
Torso, back waist	27	3.9
Multiple body parts	25	3.6
Internal organs	9	1.3

* Sudden movement of patient or patient’s relatives, carelessness by the patient or patient’s relatives, violence. ** Needle, syringe, scalpel, broken ampoule, glass items, etc. *** Electrocution, road traffic accident, heart attack.

**Table 3:** Injury types according to the characteristics of the occupational injury victims, 2020–2023.

	Sharps Injury *		Violence	
	(*n* = 339)		(*n* = 68)	
	(*n*)	(*%*) **	(*n*)	(*%*) *
**Gender**				
Female (*n* = 408)	223	54.7	29	7.1
Male (*n* = 286)	116	40.6	39	13.6
*p*-value	**<0.001**		**0.004**	
**Age groups**				
Under 30 years (*n* = 275)	157	57.1	25	9.1
30–39 years (*n* = 191)	88	46.1	21	11
40–49 years (*n* = 163)	74	45.4	15	9.2
50 years and above (*n* = 65)	20	30.8	7	10.8
*p*-value	**0.001**		0.896	
**Educational status**				
High school and below (*n* = 363)	142	39.1	29	8.0
Associate degree and above (*n* = 331)	197	59.5	39	11.8
*p*-value	**<0.001**		0.093	
**Occupation**				
Nurse (*n* = 223)	158	70.9	16	7.2
Cleaning staff (*n* = 233)	116	49.8	3	1.3
Other (*n* = 238)	65	27.3	49	20.6
*p*-value	**<0.001**		**<0.001**	
**Total working time in hospital**				
Less than 5 year (*n* = 315)	192	61.0	14	4.4
5 years and more (*n* = 379)	147	38.8	54	14.2
*p*-value	**<0.001**		**<0.001**	
**Total working time in department**				
Less than 1 year (*n* = 204)	114	55.9	10	4.9
1 year and more (*n* = 490)	225	45.9	58	11.8
*p*-value	**0.017**		**0.005**	

* Needle, syringe, scalpel, broken ampoule, glass items etc. ** Row percentage.

**Table 4:** Sharps injury types according to the characteristics of the occupational injury victims, 2020–2023.

	Needlestick		Waste Collection ^1^		Others ^2^	
	(*n*)	(*%*) *	(*n*)	(*%*) *	(*n*)	(*%*) *
**Gender**						
Female	144	64.6 ^a^	55	24.7 ^b^	24	10.8 ^b^
Male	30	25.9	57	49.1	29	25
*p*-value	**<0.001**					
**Age groups**						
Under 30 years	118	57.3 ^a^	608	29.1 ^b^	28	13.6 ^a.b^
30–39 years	56	42.1	52	39.1	25	18.8
*p*-value	**0.024**					
**Educational status**						
High school and below	21	12.6 ^a^	108	64.7 ^b^	38	22.8 ^c^
Associate degree and above	153	89	4	2.3	15	8.7
*p*-value	**<0.001**					
**Occupation**						
Nurse	154	97.5 ^a^	0	0.0 ^b^	4	2.5 ^c^
Cleaning staff	1	0.9 ^a^	106	91.4 ^b^	9	7.8 ^c^
Other	19	29.2 ^a^	6	9.2 ^a^	40	61.5 ^b^
*p*-value	**<0.001**					
**Total working time in the hospital**						
Less than 5 years	104	54.2	59	30.7	29	15.1
5 years and more	70	47.6	53	36.1	24	16.3
*p*-value	0.475					
**Total working time in the department**						
Less than 1 year	61	53.5	36	31.6	17	14.9
1 year and more	113	50.2	76	33.8	36	16
*p*-value	0.849					
**Time of the occupational injury**						
08:00–16:59	88	45.8 ^a^	60	31.3 ^a^	44	22.9 ^b^
17:00–07:59	86	58.5	52	35.4	9	6.1
*p*-value	**<0.001**					

^a^, ^b^, and ^c^ indicate statistical differences between the groups in the same row. * Row percentage. ^1^ Sharp injuries during the collection or transportation of medical sharp objects. ^2^ Others sharps: glass, scissors, laboratory materials, lancets, etc.

## Data Availability

Data and materials are available on request to authors.
